# Contactless analysis of heart rate variability during cold pressor test using radar interferometry and bidirectional LSTM networks

**DOI:** 10.1038/s41598-021-81101-1

**Published:** 2021-02-04

**Authors:** Kilin Shi, Tobias Steigleder, Sven Schellenberger, Fabian Michler, Anke Malessa, Fabian Lurz, Nicolas Rohleder, Christoph Ostgathe, Robert Weigel, Alexander Koelpin

**Affiliations:** 1 Institute for Electronics Engineering, Friedrich-Alexander-Universität Erlangen-Nürnberg (FAU), 91058 Erlangen, Germany; 2 Department of Palliative Medicine, Universitätsklinikum Erlangen, Comprehensive Cancer Center CCC Erlangen-EMN, Friedrich-Alexander-Universität Erlangen-Nürnberg (FAU), 91054 Erlangen, Germany; 3 Hamburg University of Technology, Institute of High-Frequency Technology, 21073 Hamburg, Germany; 4 Chair of Health Psychology, Friedrich-Alexander-Universität Erlangen-Nürnberg (FAU), 91052 Erlangen, Germany

**Keywords:** Diagnosis, Biomedical engineering, Electrical and electronic engineering, Cardiology

## Abstract

Contactless measurement of heart rate variability (HRV), which reflects changes of the autonomic nervous system (ANS) and provides crucial information on the health status of a person, would provide great benefits for both patients and doctors during prevention and aftercare. However, gold standard devices to record the HRV, such as the electrocardiograph, have the common disadvantage that they need permanent skin contact with the patient. Being connected to a monitoring device by cable reduces the mobility, comfort, and compliance by patients. Here, we present a contactless approach using a 24 GHz Six-Port-based radar system and an LSTM network for radar heart sound segmentation. The best scores are obtained using a two-layer bidirectional LSTM architecture. To verify the performance of the proposed system not only in a static measurement scenario but also during a dynamic change of HRV parameters, a stimulation of the ANS through a cold pressor test is integrated in the study design. A total of 638 minutes of data is gathered from 25 test subjects and is analysed extensively. High F-scores of over 95% are achieved for heartbeat detection. HRV indices such as HF norm are extracted with relative errors around 5%. Our proposed approach is capable to perform contactless and convenient HRV monitoring and is therefore suitable for long-term recordings in clinical environments and home-care scenarios.

## Introduction

Heart rate describes the number of heartbeats per minute. However, the beat of a healthy heart is not absolutely regular. Fluctuations in the time intervals of adjacent heartbeats are referred to as heart rate variability (HRV)^1,2^. This variability is linked to the neurocardiac function of the body and is generated by heart-brain interactions and by dynamic non-linear processes which are regulated by the autonomic nervous system (ANS)^3^. It is influenced by many factors such as exercise and mental stress or physiological events such as respiration, blood pressure regulation, or the circadian rhythm^4,5^. HRV is an indicator for both chronic and psychological conditions such as depression, epileptic seizures^6^, diabetic neuropathy^7^, or the surveillance of post-infarction patients to prevent sudden cardiac death^8^. Especially chronic diseases pose a major concern in future health care as both loss of quality of life and consumption of resources due to their effects are immense and their impact rises steadily.

On the other side lies a tremendous chance in addressing chronic diseases in a timely fashion as by adequate prophylactic measures, their course can be altered drastically and outcomes can be changed fundamentally. In order to achieve this, HRV needs to be measured over long periods in a way, which is both agreeable for the patient and resource-friendly for society. Established devices, which are capable to measure HRV indices, are the electrocardiograph (ECG)^2,8^ or the photoplethysmograph (PPG)^9^. However, these devices have the common disadvantage that they need permanent contact to the patient. Especially during long-term recordings, this reduces their mobility, comfort, and compliance^10^. Furthermore, electrodes might lead to further distress, false alarms and thereby increase symptom burden^11^.

A very promising approach for this task are radar systems. Radar technology promises an unobtrusive way for continuous and touch-free monitoring of vital signs^12–21^. A radar system transmits an electromagnetic wave, which is scattered at the proband’s body and received back by the radar. By evaluation of the propagation time between transmit and reception of the signal the relative distance can be determined. Another radar concept is based on phase measurements between transmit and receive signal for distance monitoring. Using this technique, researchers have reported high accuracies when detecting heart rates and single heartbeats, not only with direct line of sight but also through obstacles such as clothing. However, in order to reliably measure HRV, heart rate determination is not sufficient. Precise beat-to-beat detection and segmentation is needed to obtain correct values since small fluctuations in the detection timing have high impact on the resulting HRV indices.

Previous works that researched radar-based heart beat or HRV extraction utilised the pulse wave component as cardiac signal. However, the shape and configuration of the pulse wave is connected to respiration and therefore changes continuously during each respiration cycle. This might complicate precise heartbeat extraction that is needed for HRV determination. For the first time, this work explores the possibility of HRV extraction using the radar heart sound component, which promises higher accuracy and precision when compared to cardiac analysis using the pulse wave component due to its prominent shape and temporal distinctness^19,22^. In order to perform precise beat-to-beat localisation in the heart sound signal for HRV calculation, a hidden-Markov-based algorithm and a bidirectional Long Short-Term Memory (LSTM) network are implemented, evaluated, and compared. Several LSTM configurations are tested to obtain the optimal set of hyperparameters. Whereas previous works only evaluated the measurability and performance of radar-based HRV extraction in a static setting, e.g., in a supine or seated position, we employed a defined test scenario including a stimulation of the ANS through a cold pressor test (CPT). Thereby, the indices are not only measured at rest but also during sudden changes of the HRV parameters. To obtain a conclusive result, data from 25 test subjects are gathered and a reference ECG is used to perform an extensive evaluation of the performance of heartbeat detection and HRV extraction.

## Heart rate variability

To quantify HRV, different sets of parameters have been introduced. These indices can be separated into two major groups: Time-domain and frequency-domain parameters^2,23,24^. Time domain indices provide a quantification of the amount of variance in the inter-beat intervals (IBIs) using statistical measures. Commonly known parameters comprise the standard deviation of the NN intervals (SDNN) or the the root mean square successive difference of intervals (RMSSD). Another established parameter is the triangular index (TRI), which represents the integral of the density of the IBI histogram divided by its height^2^. TRI has a high validity^25,26^ and can be used to distinguish between normal heart rhythms and arrhythmias^27^. The major advantage of geometric measures such as TRI compared to measures like SDNN or RMSSD is their relative insensitivity to computational errors of the IBI values^28^. Therefore, in this work, TRI was selected for further analysis.

Frequency-domain measures are calculated by transforming a windowed IBI signal into frequency-domain using the discrete Fourier transform (DFT)^2^. Afterwards, the power distribution among certain frequency bands is analysed. Frequency-domain parameters are separated into high-frequency (HF) and low-frequency (LF) bands^2^. The LF band ranges from 0.04 Hz ... 0.15 Hz, equalling rhythms or modulations with periods lengths of 7 s ... 25 s. LF has often been used as a marker for sympathetic activity^29^. The HF band ranges from 0.15 Hz ... 0.4 Hz, corresponding to rhythms between 2.5 s ... 7 s. This band reflects parasympathetic or vagal activity and is sometimes also called the respiratory band since it is affected by Heart rate (HR) variations which are caused by the respiratory cycle, an effect which is called “respiratory sinus arrhythmia”. Low HF values are associated with stress, panic, anxiety, or worry. Another frequency-domain parameter is LF/HF ratio, which is simply calculated as the ratio of the respective absolute values. Since both sympathetic nervous system (SNS) and the parasympathetic nervous system (PNS) regulate HR, this value tries to reflect the dominance of either sympathetic or parasympathetic activity. Low LF/HF ratios are assumed to reflect stronger parasympathetic activity while high ratios may indicate higher sympathetic activity^1,4^.

Two measures are usually calculated for each band, the absolute power and the normalised value. The unit of the absolute power in a certain band is $\hbox {ms}^{2}$. Normalised values are calculated by dividing the respective power by the total power of LF and HF and are given in normalised units (n.u.). These values represent the percentage of the component in the total activity and emphasise the controlled and balanced behaviour of the SNS and PNS. Moreover, normalisation minimises the effect of changes in the total power which might lead to incorrect conclusions^1,2^.

## Methods

### Subjects

The university hospital in Erlangen was responsible for the acquisition and support of test subjects, the clinical validation, and acquiring the approval of the ethics committee. All of the data recordings were performed at the university hospital in Erlangen. Overall, measurements were performed with 25 healthy subjects. The written consent was obtained from all participants of this study.

Before each measurement medical staff checked the health state of the participants. The test persons were then informed in detail about the measurement procedure and were able to ask questions about any uncertainties. Before starting the measurement, the participants laid down on the examination table and were wired to the so-called “Task Force Monitor (TFM)” by *CNSystems*. This medical gold standard device was used for recording the reference ECG signal synchronised to the radar measurements.

The average age of all participants is 24.3 years with a standard deviation of 2.8 years. The average body mass index of all participants is $23.7\hbox { kg/m}^{2}$ with a standard deviation of $2.9\hbox { kg/m}^{2}$. Equal gender ratio is observed (female $n=12$, male $n=13$).

### Measurement setup and synchronisation

**Figure 1:**
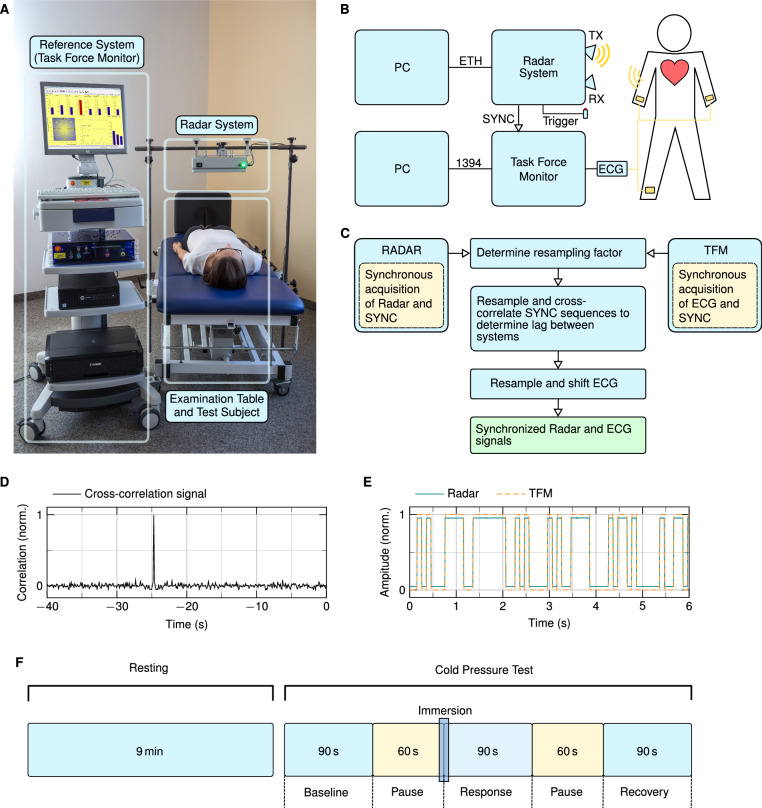
Overview of the measurement setup and measurement protocol. (**A**) Photograph of the measurement setup, including the TFM, the radar system, and the examination table^17^. (**B**) Block diagram of the measurement setup. (**C**) Flowchart of the data synchronisation routine. (**D**) Cross-correlation of the SYNC sequences which is used to determine the latency between both systems. (**E**) Synchronisation sequences of the TFM and the radar system after resampling and time shift removal. (**F**) Depiction of the measurement protocol: after a resting measurement of nine minutes, the CPT manoeuvre is performed, which includes three main parts with a length of 90 s and pauses with a length of 60 s in between.

A picture of the measurement setup can be seen in Fig. 1A. The radar system is mounted on a fixed construction placed above the examination table and focuses on the chest area of the test subjects. The height above the chest as well as the position of the radar can be adapted. On average, the distance between the antenna and the skin of a test subject was around 40 cm. A block diagram of the setup is depicted in Fig. 1B. The signals of the radar system and the TFM are sampled by two different computers. The signals are synchronised during post-processing by creating a sequential binary synchronisation sequence according to the Gold codes inside the radar system. These sequences are sampled by the radar system as well as the reference device by feeding the synchronisation sequence into the TFM by connecting a cable to the external input. The synchronisation procedure is shown in Fig. 1C. Since the radar system and the TFM system have different sampling frequencies, the first step is to find the resampling factor that is used to obtain the same sampling rate. This is done by optimising the factor using the fminbnd function in MATLAB and taking the maximum cross-correlation value as objective function. This resampling factor is used to determine the latency between ECG and radar signal by searching for the maximum in the cross-correlation of both synchronisation sequences as shown in Fig. 1D. The time shift results from the asynchronous start of both systems. Using both the resampling factor and the latency, the ECG signal is resampled and shifted in order to obtain synchronised radar and ECG signals. A section of a resampled and shifted synchronisation sequence of both systems is depicted in Fig. 1E. After synchronisation, both signals have a sample rate of 2000 samples per second.

### Cold pressor test

The goal of this study is to examine the possibility of contactless HRV measurement using radar technology. For this purpose, a test procedure analyses HRV parameters of healthy test subjects simultaneously collected by both a reference ECG and the radar system. In order to achieve a reliable statement whether the radar is capable of accurately determining the parameters not only in a static setting but also in the case of abrupt HRV changes, the designed test protocol includes not only a resting scenario but also a forced activation of the ANS. In order to force a stimulation of the ANS, the CPT was chosen as part of the experimental protocol. The CPT is a test procedure designed to provoke a sympathetic activation and an increase in blood pressure in healthy subjects^30,31^. The CPT manoeuvre is typically performed by immersing a subject’s hand into ice water ($1{^{\circ }}\hbox {C}\, ... \, 5{^{\circ }}\hbox {C}$) for a short period of several minutes (1 min ... 6 min) or as long as possible^30^.

### Experimental protocol

The measurement routine is depicted in Fig. 1F. First, a resting measurement was performed for nine minutes. During this time, the test subject was asked to lie calmly on the examination table. Room temperature and brightness were set to a comfortable level. The resting measurement ensures the test subjects for being able to calm down in order to have a comparable starting point.

Next, the CPT sequence was started. The data gathered from these recordings are used to compare the extracted HRV values of the reference device and the radar system to each other. The CPT manoeuvre was split into three parts of equal length: “Baseline”, “Response”, and “Recovery” with pauses of 60 s in between. HRV comparison is performed separately for all three parts. Baseline serves as a reference starting point, Response measures the abrupt change of HRV during the ice water stimulation, and Recovery provides information about the ability to return to normal condition. These windows were chosen in order to achieve large amount of variation between the successive HRV values since the overall objective is to evaluate the ability of radar systems to correctly determine HRV values not only in one setting but also after abrupt changes.

Each of the three parts has a length of 90 s. This length was chosen due to practical and theoretical considerations. During Response, the test subjects were asked to hold their hands into the ice water for two minutes or as long as possible. The windowed data which are used for the HRV calculation of this part, however, should be fully covered by this action, i.e., the window length should not be longer than 120 s. A slightly shorter window would be preferred in case a subject is not able to hold their hands into the ice water for the entire two minutes. Although the standard window length of short-time HRV measurements is five minutes, researchers have shown that the calculation of valid HRV parameters is possible for shorter windows^3,32,33^. Parameters such as HF, LF, or LF/HF ratio can be calculated for a window length of 90 s, therefore, this length was chosen as overall window length for HRV calculation. It is important to choose the same window length for all parts of the CPT manoeuvre in order to ensure comparability^2^.

The overall measurement of the CPT sequence is started a little ahead of Baseline and is stopped a little behind Recovery, in order to make sure that the same depicted sequence can be extracted from every test subject. “Immersion” represents the point in time at which the subject is asked to put the hand into the ice water. Removing the hand from the water happens after a maximum of two minutes or earlier, so this point in time may lie after the end of the Response window but never in the Recovery part.

### Six-Port interferometry

The Six-Port consists of two input and four output ports. The input signals are the reference signal $P_1$ and the signal $P_2$ which is the signal that is reflected at the target and received by the antenna. In the Six-Port structure, these two signals are superimposed under four relative and static phase shifts of $0{^{\circ }}, 90{^{\circ }}, 180{^{\circ }}$, and $270{^{\circ }}$. These four output signals $P_{3\,...\,6}$ are then down-converted into baseband using diode power detectors, resulting in baseband signals $B_{3\,...\,6}$. The baseband signals form two pairs of differential signal which represent the orthogonal in-phase and quadrature components *I* and *Q* of a complex number ${\underline{Z}}$^34^:

1 $$\begin{aligned} {\underline{Z}} = I + jQ = (B_5 - B_6) + j(B_3 - B_4). \end{aligned}$$

The relative distance change $\Delta x$ can then be extracted from ${\underline{Z}}$ using:

2 $$\begin{aligned} \Delta x = \frac{\Delta \varphi }{2\pi } \cdot \frac{\lambda }{2}, \end{aligned}$$

with $\lambda$ being the known wavelength of the signal and $\Delta \varphi$, the argument of ${\underline{Z}}$, representing the relative phase shift between the two input signals:

3 $$\begin{aligned} \Delta \varphi = \text {arg}\{{\underline{Z}}\} = \text {arg}\{(B_5 - B_6) + j(B_3 - B_4)\}. \end{aligned}$$

### Calculation and processing of HRV parameters

The HRV parameters HF, LF, LF/HF ratio, and TRI are calculated for both the radar and the ECG signal. The first step hereby is to extract single heartbeats from the ECG and the radar heart sound signal. For the ECG, the R-peaks are used as reference points which are located using the algorithm presented by Zhang et al.^35^. Afterwards, detected peaks are manually inspected to check for erroneous labels. The interval between two successive heartbeats is called RR interval whereas a set of successive RR intervals is called RR sequence. To obtain radar RR sequences, the radar heart sound signals are segmented first. The start of the first state, i.e., the first heart sound, is used as reference point for each heartbeat of the radar heart sound signal.

The RR sequences of the ECG and radar signal are checked for invalid values using an automated procedure. These correction steps are important since single outliers will otherwise cause large errors in the resulting HRV parameters, especially when calculating frequency-domain parameters^2^. To remove artefacts, e.g., missed beats or falsely detected beats, an algorithm for artefact removal is utilised which is based on the work by Vollmer^36,37^. After artefact removal, the HRV parameters are calculated. Time-domain parameters can be directly derived from the RR sequences. To calculate frequency-domain parameters like LF or HF, the RR sequences are linearly interpolated and standardized first. Interpolation is necessary since equidistant sampling is required in order to calculate the DFT. Before calculating the DFT, the signal is zero-padded until its length is a power of two.

### Human subjects

The study was approved by the ethics committee of the Friedrich-Alexander-Universität Erlangen-Nürnberg (No. 85_15B). All research was performed in accordance with relevant guidelines and regulations. The informed consent was obtained from all subjects in human trials.

### Contactless heart sound recording using radar interferometry

The first step of contactless HRV acquisition is to measure cardiac vital signs using radar. Two approaches are possible, either to measure the motion of the chest surface due to the propagation of the pulse waves^13,15,18,38,39^ or to record the substantially smaller vibrations of the skin due to the heart sounds^19,40–43^. The latter technique has proved to deliver higher accuracies when determining the timings of the single heartbeats^19^. This is preferable since HRV represents the variation of the time intervals of single successive heartbeats. Precisely locating single heartbeats is therefore essential since outliers cause large errors in the resulting HRV values, especially when calculating frequency-domain parameters^2^.

**Figure 2:**
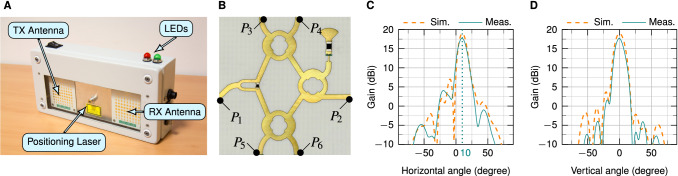
The radar system and its components^17^. (**A**) Photograph of the radar system. (**B**) Planar realisation of the Six-Port structure. (**C**) Simulated (Sim.) and measured (Meas.) radiation pattern in horizontal plane. (**D**) Simulated and measured radiation pattern in vertical plane at $10{^{\circ }}$ inclination.

Six-Port-based interferometers have shown to be able to measure heart sound signals using a phase evaluation technique^34,44^. In this study, a bistatic 24 GHz Six-Port-based radar system was built and utilised which can be seen in Fig. 2A. A photo of the Six-Port structure is shown in Fig. 2B. The system consists of two antennas, a transmitting (TX) as well as a receiving (RX) antenna. The beams of the antennas are tilted towards a common focal point at a target distance of around 40 cm^17^. The resulting inclination angle of the antennas is $\pm 10{^{\circ }}$. The antennas have a simulated gain of 19 dBi and a measured gain of 17.7 dBi each and are realised using a planar design in order to facilitate the integration into the housing. The 72 patches of the antenna are arranged in nine columns with eight elements each. The simulated and measured radiation patterns of the antennas in both horizontal and vertical plane are shown in Fig. 2C,D. It can be seen that measured and simulated value exhibit high agreement. The system has an equivalent isotropically radiated power (EIRP) smaller than 20 dBm, which is the limit as defined by the regulations for the industrial, scientific, and medical (ISM) band.

### Algorithms for heart sound segmentation

An LSTM network and a hidden semi-Markov model (HSMM)^45^ are used for the detection of single heartbeats in the radar heart sound signal. The HSMM model is implemented as described in^19^. Three features are used for training and testing: the homomorphic, the Hilbert, and the power spectral density envelope of the heart sound signal. The HSMM is chosen as state-of-the-art algorithm since it is the only segmentation technique that has been evaluated for radar heart sound analysis.

However, the HSMM algorithm has the disadvantage that it requires a priori information. Due to its limited heart rate range, it sometimes lacks the capability to correctly detect high variations of the heart rate. Recurrent neural network (RNNs) architectures and especially LSTMs promise to be a fitting candidate to overcome these drawbacks. LSTM networks are a special type of RNN, which are able to learn time-dependencies. Hochreiter and Schmidhuber^46^ introduced LSTMs to avoid long-term dependency problems and handle issues that conventional RNNs face. Standard LSTMs can only take the preceding elements into account. However, for the task of heart sound segmentation, subsequent inputs might also carry relevant information regarding the state change^47^. Therefore, bidirectional LSTMs are employed in this work, which consist of two LSTM layers. One layer computes the forward embedding while the other layer computes the backward layer. The final result is obtained by concatenating both embeddings.

Several LSTM configurations were thoroughly evaluated for the purpose of radar heart sound segmentation using leave-one-subject-out cross-validation (LOSOCV). The models are hereby trained on the data of all subjects except the one that is tested. This is iterated until the data of all subjects have been tested once. Models with one and two LSTM or bidirectional LSTM layers are compared. Furthermore, the units per layer are varied between 50 and 400 hidden units. The best results are obtained using a model with two bidirectional LSTM layers containing 400 hidden units in the first layer and 200 hidden units in the second layer. A dropout layer with a drop probability of 0.2 is used after each LSTM layer to combat overfitting. The LSTM layers are followed by a fully-connected layer, a softmax layer, and a classification layer. Training is done for 50 epochs with a batch size of 64. The initial learn rate is 0.001 and drops after every 20 epochs by a factor of 10. The utilised features include the homomorphic, the Hilbert, and the power spectral density envelope of the heart sound signal as for the HSMM model. Additionally, heart rate is estimated using the autocorrelation of the heart sound signal. This is done by searching for the maximum in the autocorrelation signal in a certain range, corresponding to heart rates of 40 to 135 beats per minute (bpm). This value is used as a feature for every time step of the windowed signal that is segmented.

## Results

### Performance evaluation of beat-to-beat detection and HRV extraction

To determine the performance of the radar system for HRV analysis, the first step is to evaluate the scores when detecting single heartbeats. A reference heartbeat is defined at every R-peak in the ECG signal. This point in time also corresponds to the start of the first heart sound. LSTM and HSMM return a segmentation of the heart sound signal consisting of four states: first heart sound, systole, second heart sound, and diastole. To compare the performance of heartbeat extraction and HRV calculation, the start of each first state, i.e., each detected first heart sound, is used for comparison.

Scores are obtained using LOSOCV. Four figures are compared: F1 score, sensitivity, precision, and accuracy. Sensitivity is calculated as the number of true positives (TP) divided by the sum of true positives and false negatives (FN). Precision is defined as the number of true positives divided by the sum of true positives and false positives (FP). Accuracy is calculated as the number of true positives divided by the sum of all true positives, false positives, and false negatives. The F1 score represents a harmonic mean of sensitivity and precision and can be calculated as: $(2\cdot \text {TP}) / (2\cdot \text {TP}\cdot \text {FP}\cdot \text {FN})$.

A detected heartbeat is counted as true positive when it deviates no more than 75 ms from the reference heartbeat. This corresponds to half of the recognised tolerance range of 150 ms for ECG R-peak detection^48^. A false negative is counted when no heartbeat is detected within this range around an ECG R-peak. A false positive is added when there is no ECG R-peak around a detected one. The scores for all test subjects, separated by the nine minutes resting measurement and the CPT manoeuvre, are summarised in Table 1. In addition to the mean score of the whole CPT manoeuvre, the scores for the three single CPT scenarios, Baseline, Response, and Recovery, are also shown. The data from subject 20 was excluded due to massive clipping of the baseband signals. For the resting measurement, the LSTM achieves an F1 score of 99.01%, a sensitivity of 98.89%, a precision of 98.94%, and an accuracy of 97.86%. The HSMM scores four to eight percentage points lower in comparison with an F1 score of 93.67%, a sensitivity of 94.35%, a precision of 94.62%, and an accuracy of 89.54%. For the CPT manoeuvre, the LSTM also scores around three to seven percentage points higher in comparison, with an F1 score of 96.28%, a sensitivity of 95.54%, a precision of 96.01%, and an accuracy of 91.89%. It can be noted that the resting scores are on average higher than the CPT scores. This is due to the lower scores during the Recovery scenario. Overall, higher scores can be observed for the LSTM algorithm for all figures.

**Table 1:** Performance comparison of LSTM and HSMM for single heartbeat detection.

	F1					Sensitivity				
	Resting (%)	CPT				Resting (%)	CPT			
		Baseline (%)	Response (%)	Recovery (%)	Mean (%)		Baseline (%)	Response (%)	Recovery (%)	Mean (%)
LSTM	99.01	97.76	92.98	98.09	96.28	98.89	97.96	91.39	97.70	95.54
HSMM	93.67	94.48	86.57	94.19	91.75	94.35	95.01	88.18	93.96	92.25

	Precision					Accuracy				
	Resting (%)	CPT				Resting (%)	CPT			
		Baseline (%)	Response (%)	Recovery (%)	Mean (%)		Baseline (%)	Response (%)	Recovery (%)	Mean (%)
LSTM	98.94	97.33	92.92	98.05	96.01	97.86	95.39	85.44	95.84	91.89
HSMM	94.62	95.40	85.02	94.49	91.33	89.54	90.85	76.32	89.08	84.82

High scores in Table 1 indicate that HRV can in principle be measured by radar. To further validate this assumption, LSTM and HSMM performances are evaluated for the calculation of several HRV parameters. Besides the data from test subject 20, which was excluded due to clipping of the radar signal, the data from test subjects 8 and 16 also had to be excluded from HRV calculation. Test subject 8 was excluded since she was not able to hold their hand in the ice water for a sufficient time and test subject 16 was excluded since no trigger signals were acquired during the measurement, i.e., the single parts of the CPT could not be distinguished afterwards. Several HRV parameters are compared: Heart rate (HR), HF, HF norm, LF, LF/HR ratio, and TRI. Although the heart rate is not a typical HRV parameter per se, it is closely linked to HRV and is also widely used for medical decision-making.

**Table 2:** Comparison of calculated HRV parameters.

			Group neg. (mean ± std.dev.)			Group pos. (mean ± std.dev.)			Overall (percentage difference)	
			ECG	LSTM	LSTM	ECG	LSTM	HSMM	Difference $\hbox{LSTM} \leftrightarrow \hbox{ECG} (\%)$	Difference $\hbox{HSMM} \leftrightarrow \hbox{ECG} \,(\%)$
**Heart Rate (bpm)**	Resting		$55.81\pm 5.90$	$55.80\pm 5.86$	$55.71\pm 5.89$	$61.56\pm 11.60$	$61.55\pm 11.61$	$61.42\pm 11.48$	**0.16**	**0.35**
	CPT	Baseline	$56.78\pm 7.59$	$56.79\pm 7.60$	$56.79\pm 7.61$	$62.22\pm 11.70$	$62.61\pm 11.94$	$61.92\pm 11.84$	0.79	0.45
		Response	$63.26\pm 11.73$	$62.80\pm 10.81$	$62.88\pm 11.96$	$67.12\pm 13.11$	$65.24\pm 11.69$	$70.67\pm 17.84$	1.98	5.27
		Recovery	$55.79\pm 5.99$	$55.90\pm 6.06$	$55.92\pm 6.06$	$60.56\pm 10.84$	$60.65\pm 11.18$	$60.19\pm 10.69$	0.41	0.41
		Mean	$58.61\pm 9.38$	$58.49\pm 8.93$	$58.53\pm 9.43$	$63.30\pm 12.24$	$62.83\pm 11.76$	$64.26\pm 14.56$	**1.06**	**2.08**
**HF (stand.)**	Resting		$4.45\pm 0.66$	$4.55\pm 0.64$	$4.70\pm 0.59$	$3.91\pm 1.06$	$4.14\pm 1.07$	$4.24\pm 0.91$	**8.17**	**14.74**
	CPT	Baseline	$4.66\pm 0.51$	$4.87\pm 0.58$	$5.02\pm 0.72$	$3.61\pm 1.15$	$3.73\pm 1.07$	$3.96\pm 0.89$	11.39	21.68
		Response	$4.14\pm 0.72$	$4.21\pm 1.06$	$4.20\pm 0.54$	$3.84\pm 1.27$	$4.07\pm 1.17$	$4.24\pm 1.32$	10.04	11.75
		Recovery	$4.91\pm 0.92$	$5.39\pm 0.89$	$5.33\pm 0.96$	$3.87\pm 1.34$	$4.11\pm 1.21$	$4.45\pm 1.25$	11.19	17.88
		Mean	$4.57\pm 0.80$	$4.82\pm 0.99$	$4.85\pm 0.90$	$3.77\pm 1.26$	$3.97\pm 1.17$	$4.21\pm 1.19$	**10.87**	**17.10**
**HF norm (n.u.)**	Resting		$61.19\pm 6.34$	$61.80\pm 6.29$	$62.31\pm 6.35$	$52.05\pm 10.38$	$53.61\pm 10.40$	$53.92\pm 8.25$	**4.83**	**8.68**
	CPT	Baseline	$64.60\pm 8.01$	$65.86\pm 7.32$	$66.74\pm 7.69$	$47.29\pm 10.33$	$48.83\pm 9.15$	$49.71\pm 8.25$	5.26	12.99
		Response	$57.88\pm 5.26$	$55.60\pm 5.46$	$56.58\pm 5.47$	$55.58\pm 10.44$	$54.25\pm 9.18$	$56.42\pm 7.65$	4.96	5.45
		Recovery	$61.62\pm 8.23$	$66.56\pm 8.51$	$62.63\pm 6.80$	$51.34\pm 13.49$	$52.75\pm 13.23$	$55.68\pm 12.43$	6.36	10.80
		Mean	$61.37\pm 7.79$	$62.67\pm 8.78$	$61.98\pm 7.90$	$51.40\pm 12.00$	$51.94\pm 10.93$	$53.94\pm 10.13$	**5.52**	**9.75**
**LF (stand.)**	Resting		$2.84\pm 0.64$	$2.84\pm 0.62$	$2.88\pm 0.56$	$3.49\pm 0.57$	$3.47\pm 0.58$	$3.55\pm 0.51$	**4.82**	**7.48**
	CPT	Baseline	$2.60\pm 0.78$	$2.55\pm 0.70$	$2.53\pm 0.75$	$3.86\pm 0.51$	$3.78\pm 0.66$	$3.95\pm 0.60$	5.86	7.08
		Response	$3.00\pm 0.52$	$3.31\pm 0.59$	$3.25\pm 0.63$	$2.93\pm 0.70$	$3.34\pm 0.72$	$3.14\pm 0.58$	16.26	11.79
		Recovery	$3.05\pm 0.68$	$2.70\pm 0.71$	$3.13\pm 0.49$	$3.57\pm 0.93$	$3.62\pm 0.97$	$3.47\pm 0.89$	8.61	12.43
		Mean	$2.88\pm 0.70$	$2.85\pm 0.75$	$2.97\pm 0.71$	$3.45\pm 0.83$	$3.58\pm 0.81$	$3.52\pm 0.78$	**10.25**	**10.43**
**LF/HF ratio**	Resting		$0.67\pm 0.18$	$0.65\pm 0.18$	$0.65\pm 0.17$	$1.04\pm 0.45$	$0.98\pm 0.44$	$0.93\pm 0.28$	**9.43**	**16.02**
	CPT	Baseline	$0.57\pm 0.21$	$0.54\pm 0.19$	$0.52\pm 0.21$	$1.23\pm 0.52$	$1.13\pm 0.44$	$1.06\pm 0.32$	8.80	21.61
		Response	$0.74\pm 0.17$	$0.81\pm 0.17$	$0.78\pm 0.17$	$0.87\pm 0.38$	$0.90\pm 0.37$	$0.81\pm 0.25$	13.42	11.30
		Recovery	$0.65\pm 0.20$	$0.53\pm 0.20$	$0.61\pm 0.17$	$1.08\pm 0.53$	$1.02\pm 0.50$	$0.89\pm 0.44$	11.22	21.44
		Mean	$0.66\pm 0.21$	$0.63\pm 0.23$	$0.64\pm 0.21$	$1.06\pm 0.51$	$1.02\pm 0.45$	$0.92\pm 0.36$	**11.15**	**18.12**
**TRI**	Resting		$11.52\pm 2.93$	$10.19\pm 2.21$	$12.39\pm 2.67$	$11.13\pm 2.75$	$10.17\pm 2.67$	$11.41\pm 3.02$	**17.67**	**17.43**
	CPT	Baseline	$11.08\pm 2.26$	$9.45\pm 3.06$	$12.75\pm 2.82$	$11.09\pm 2.35$	$11.02\pm 2.76$	$11.85\pm 4.27$	12.92	18.97
		Response	$12.85\pm 6.26$	$12.06\pm 4.15$	$14.43\pm 3.56$	$12.01\pm 2.87$	$10.48\pm 2.69$	$12.70\pm 3.59$	14.45	24.16
		Recovery	$10.48\pm 2.38$	$8.58\pm 1.59$	$11.00\pm 1.37$	$10.03\pm 2.52$	$9.49\pm 2.63$	$10.72\pm 2.97$	23.15	11.97
		Mean	$11.47\pm 4.20$	$10.03\pm 3.45$	$12.73\pm 3.07$	$11.04\pm 2.71$	$10.33\pm 2.77$	$11.76\pm 3.74$	**16.84**	**18.37**

The HRV parameters of the reference ECG are compared to the radar HRV parameters using both the LSTM and HSMM algorithm. The absolute values are compared for the two groups (mean ± standard deviation), while the mean deviation of the parameters are given for all test subjects.

Table 2 compares the HRV indices that are obtained using the radar system and the LSTM/HSMM algorithm to the reference ECG HRV indices. Rows correspond to different parameters and scenarios while columns represent the methods. The first row corresponds to the nine-minute resting measurement (Rest.) while the other rows represent the CPT manoeuvre. The CPT values are again split into the three scenarios Baseline (Base.), Response (Resp.), and Recovery (Reco.) as well as a mean score. The first six columns display absolute values with their mean and standard deviation. The results from different test subjects are separated into two groups. The groups were formed by distinguishing the different reactions of the test subjects to the CPT. As noted by Mourot  et al.^30^, some people react with an increasing HF norm in response to ice water stimulus while others exhibit a decreasing HF norm. Therefore, the scores of the test subjects are separated under this consideration. Test subjects 1, 4, 6, 7, 12, and 25 had a decrease of HF norm due to the ice water stimulus, i.e., a lower HF norm during Response in comparison to Baseline. Their scores are summarised under “Group neg.” while the others are summarised under “Group pos.”. The inverse reactions of different test subjects are no drawback for this study as the direction of change of the HRV parameters is not important. The goal of this study is to demonstrate reliable measurability of HRV parameters using radar interferometry during sudden changes, therefore, only the absolute change is of interest. The table was split into two groups to underline the changes of parameters which would otherwise be hidden due to two opposing groups.

For both groups, an increase of heart rate can be observed during Response. Per definition, the negative group has a decrease of HF and HF norm during Response while the positive group has an increase of these parameters. During Recovery, HF norm return to Baseline level. As expected, LF and LF/HF ratio increase during Response in the negative group while they decrease in the positive group. Again, they return to normal level during Recovery. TRI increases in both groups during Response and decreases again during Recovery. These trends are reproduced by both the LSTM and the HSMM.

The last two columns show the percentage differences of the LSTM or HSMM values in comparison to the reference ECG values. These percentage differences are calculated by taking the mean difference over all test subjects. For HR, both LSTM and HSMM achieve deviations of 0.16%% and 0.35% for the resting measurement and 1.06% and 2.08% for the whole CPT manoeuvre. For HF, LSTM achieves substantially lower deviations of 8.17% and 10.87% in comparison to HSMM with 14.74% and 17.10%. For HF norm, LSTM again achieves small deviations of 4.83% and 5.52%, which are around four percentage points lower in comparison to the HSMM values. For LF, LSTM and HSMM have similar CPT differences of 10.25% and 10.43%, respectively. For LF/HF ratio, the differences of the LSTM algorithm are around 7% lower in comparison to HSMM, with deviations of 9.43% for the resting measurement and 11.15% for the CPT manoeuvre. For TRI, HSMM achieves a slightly lower deviation during the resting measurement but a higher deviation of 18.37% during the CPT manoeuvre.

**Figure 3:**
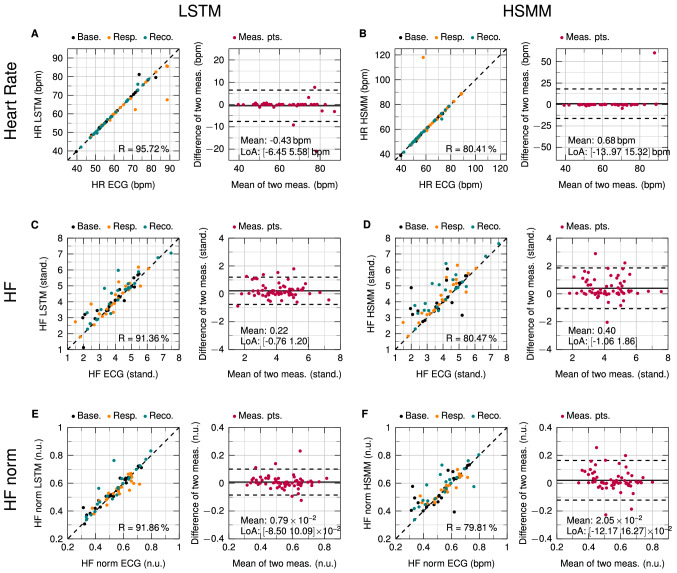
Scatterplots and Bland–Altman analyses of the parameters heart rate, HF, and HF norm. (**A**,**C**,**E**) Comparison of LSTM values with reference ECG. (**B**,**D**,**F**) Comparison of HSMM values with reference ECG.

**Figure 4:**
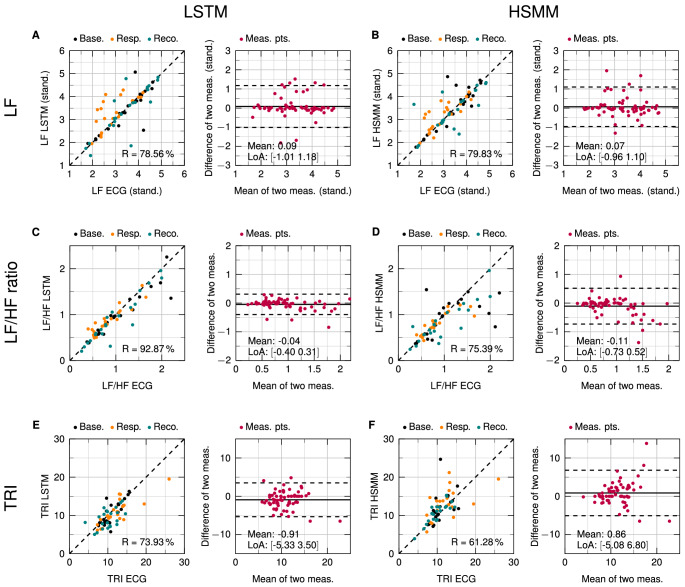
Scatterplots and Bland–Altman analyses of the parameters LF, LF/HF ratio, and TRI. (**A**,**C**,**E**) Comparison of LSTM values with reference ECG. (**B**,**D**,**F**) Comparison of HSMM values with reference ECG.

Figures 3 and 4 show scatter plots and Bland–Altman plots for heart rate, HF, HF norm, LF, LF/HF ratio, and TRI. The left and right side depict the results obtained by LSTM and HSMM, respectively. Each row corresponds to one parameter. In order to evaluate the performance of the radar under sudden HRV changes, the resting measurements were not included in the plots. The CPT measurement data in the scatter plots are visually split into the three scenarios Baseline, Response, and Recovery.

The scatter plots reveal the amount of correlation between two methods. Correlation is calculated as Pearson correlation coefficient (R). However, a high correlation does not necessarily lead to a good agreement between those methods^49^. This is the reason why Bland and Altman introduced the so-called Bland–Altman plot^50^. This plot allows for easy visual identification of any systematic errors such as a fixed or proportional bias. The mean value shows if there is any fixed bias between two methods. A fixed bias indicates that there is a constant offset between two methods while a proportional bias shows that differences between the two methods depend on the absolute value. The limits of agreement (LoA) describe a range of agreement within which 95% of the differences between the two methods are included. Small values of mean and LoA show a high agreement between two methods.

For heart rate, LSTM achieves a correlation coefficient of 95.72%. A small mean value of −0.43 bpm is observed in the Bland–Altman plot with the LoA lying at −6.45 bpm and −5.58 bpm. No proportional bias can be seen. HSMM has a correlation of 80.41%, mainly due to one outlier. The mean values of the differences is at 0.68 bpm and the LoA are at −13.97 bpm and 15.32 bpm. Compared to LSTM, the mean value as well as the LoA are substantially higher. For HF, LSTM again has a substantially higher correlation than HSMM with $\text {R} = 91.36 \%$ for LSTM and $\text {R} = 80.47 \%$ for HSMM. LSTM has a mean difference of 0.22 with the LoA at −0.76 and 1.20 while HSMM has a mean difference of 0.40 with the LoA at −1.06 and 1.86. For HF norm, similar values can be observed for R. For the LSTM, $\text {R} = 91.86 \%$ while for the HSMM, $\text {R} =79.81 \%$. In the Bland–Altman plot, a mean difference of $0.79\times 10^{-2}$ and LoA of $-8.50\times 10^{-2}$ and $10.09\times 10^{-2}$ can be observed for the LSTM. HSMM has a mean difference of $2.05\times 10^{-2}$ and LoA at $-12.17\times 10^{-2}$ and $16.27\times 10^{-2}$.

For LF, LSTM has a slightly smaller correlation of 78.56% in comparison to HSMM with a correlation of 79.83%. The mean difference of LSTM is at 0.09 with the LoA at −1.01 and 1.18. These numbers are also slightly higher than for HSMM, which has a mean difference of 0.07 and LoA at −0.96 and 1.10. For LF/HF ratio, LSTM again has a high correlation of 92.87 % with the mean difference at −0.04 and the LoA at −0.40 and 0.31. HSMM only reaches a lower correlation of 75.39% with the mean difference at −0.11 and the LoA at −0.73 and 0.52. For the time-domain parameter TRI, LSTM achieves a correlation of 73.93% in comparison to the ECG. HSMM has a correlation of 61.28%. No proportional biases can be observed in any of the Bland–Altman plots of all six parameters.

**Figure 5:**
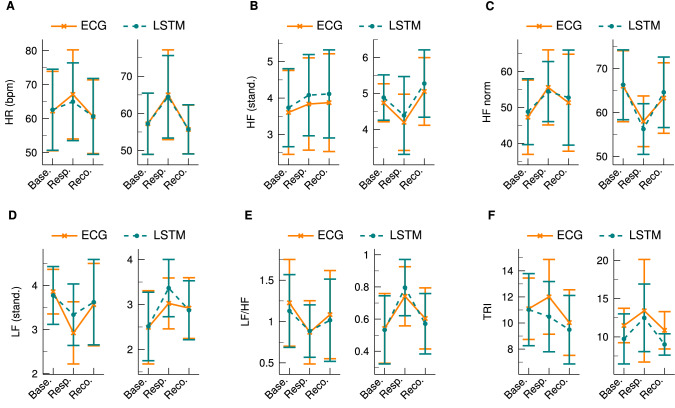
Changes of the HRV parameters before, during, and after the CPT. The plots on the left sides show the values of the positive group whereas the plots on the right sides illustrate the results of the negative group. For each scenario, the mean and standard deviation is given for both the ECG and the LSTM. (**A**) Heart rate. (**B**) HF. (**C**) HF norm. (**D**) LF. (**E**) LF/HF ratio. (**F**) TRI.

The LSTM algorithm has generally achieved better results than the HSMM model. It might be additionally of interest to investigate the dynamic changes of the parameters to determine if the LSTM is also able to reproduce the instantaneous variations in response to the stimulation of the CPT. Figure 5 shows a comparison of the HRV parameters before, during, and after the CPT. Depicted are the mean and standard deviation for each scenario and each parameter as obtained by the ECG and the LSTM. To emphasise the absolute change of the parameters in response to the CPT, the data is again split into the positive and negative group. Although there are small bias errors at Response for LF and for the negative group of TRI, the direction of change can be reconstructed for all parameters. For the positive group, a small error occurs at TRI but only for the Response scenario. In summary, the LSTM is able to demonstrate its ability to closely track the changes of the HRV parameters that are induced by the CPT.

## Discussion

We have introduced and validated a method for contactless HRV acquisition using a Six-Port-based radar system and a bidirectional LSTM model. Compared to other contactless ways of vital sign and HRV measurement such as cameras^51^ or laser systems^52^, radar technology has the advantage that it is independent of surrounding light and is able to penetrate clothing or other visually non-transparent obstacles.

The possibility of extracting HRV parameters using radar technology has been investigated in general^53–57^. Previous works focused mainly on the measurability of HRV indices in a static setting, e.g., when lying or sitting at rest. However, it has not been investigated if the systems are able to track rapidly changing HRV parameters in a dynamic setting. To research this aspect, we defined an experimental protocol including not only a resting measurement but also a subsequent CPT manoeuvre, a commonly used cardiovascular test, which was used to force a sudden change of HRV due to the stimulus of the ANS. We utilised a bistatic radar system to acquire vital sign data from 25 test subjects, a larger number of test subjects compared to previous studies. Altogether, 683 minutes of data were gathered and analysed extensively. An ECG signal was acquired in parallel which served as a reference gold standard device for heartbeat detection and HRV calculation.

Previous works utilised the pulse wave component to extract HRV. However, this may complicate precise beat-to-beat localisation due to the influence of respiration on the shape and configuration of the pulse wave. In this paper, we firstly evaluated the possibility of HRV extraction using radar heart sound signals. This promises for higher accuracies since the shape and configuration of the heart sound signal is more distinct and not influenced by respiration. Radar heart sound data were used to determine the RR sequences and HRV parameters for the radar system. We implemented two algorithms for the task of heartbeat segmentation and beat-to-beat recognition, a state of the art HSMM algorithm and a bidirectional LSTM network. Several configurations of the LSTM model were compared using different numbers of layers and hidden units. A two-layer bidirectional LSTM with 400 and 200 hidden units in the first and second layer demonstrated the best results.

For heartbeat detection, high F1 scores, sensitivities, precisions, and accuracies around 90% ... 99% have been observed for both algorithms, however, the LSTM algorithm performed around three to seven percentage points better than the HSMM. Furthermore, HRV indices of the radar system were compared to ECG HRV indices. Overall, LSTM performed better for both the resting scenario as well as the CPT manoeuvre. When acquiring parameters such as HF norm or LF/HF ratio, LSTM achieved low deviations of 5.52% and 11.15%, respectively, for the CPT, while the HSMM had higher deviations of 9.75% and 18.12%. Using scatter and Bland–Altman plots to compare radar and ECG HRV indices, correlations over 90% could be observed with small offsets and no proportional biases. We demonstrated that the LSTM algorithm is capable to perform precise heart sound segmentation in order to track the dynamic changes of HRV before, during, and after the CPT. Our results show that the proposed system is able to track HRV parameters not only in a static resting scenario but also during sudden changes.

The main benefit of our proposed system is its ability to perform contactless and accurate monitoring of HRV. Without the need of permanent cabling, this method allows for burden-free long-term monitoring which is crucial for a medical diagnosis. One of the limitations of this approach is that it is only suited for stationary usage, e.g., to monitor a person in bed. If the person leaves the bed, the radar will not be able to track the subject. However, for most use cases, this would not be considered a drawback, e.g., for patient monitoring at the hospital or for measurements at night in a domestic scenario. Another limitation of our system is random body movement. During the movement of the subject, heart sound data cannot be measured with high accuracy. Therefore, HRV cannot be determined for this period of time. Nonetheless, this is no drawback for the scenario that the system is designed for. During a long-term measurement, e.g., at night, short periods without reliable data are negligible. Sections containing low quality heart sound signals need to be automatically detected and discarded from the HRV analysis.

Our presented approach enables precise, touch-free, and continuous monitoring of HRV parameters using a 24 GHz radar system and an LSTM network. The proposed bidirectional LSTM architecture is able to perform real-time analysis of HRV parameters. This allows for a convenient way of long-term monitoring in a wide range of everyday applications. In hospitals, where latex or cold foam mattresses are commonly used, the system can be mounted under the mattress without causing any major impact, provided that the slatted frame has a gap or does not contribute significantly to damping^58^. Mounting the system above the patient is also possible, since common blankets can be easily penetrated. Additionally, its low output power, which lies within the limits of the regulations of the ISM band, makes it suitable for long-term monitoring applications not only in a clinical environment, e.g., for patient monitoring at the general ward, but also in a home-care scenario, e.g., for aftercare treatment or preventative health care.

## Data Availability

The data that supports the findings of this study are available upon request from the corresponding author.
